# Mitigating non-genetic resistance to checkpoint inhibition based on multiple states of immune exhaustion

**DOI:** 10.1038/s41540-024-00336-6

**Published:** 2024-02-09

**Authors:** Irina Kareva, Jana L. Gevertz

**Affiliations:** 1 Quantitative Pharmacology Department, EMD Serono, Merck KGaA, Billerica, MA USA; 2 Department of Biomedical Engineering, Northeastern University, Boston, MA USA; 3 Department of Mathematics and Statistics, The College of New Jersey, Ewing, NJ USA

**Keywords:** Cancer, Dynamical systems

## Abstract

Despite the revolutionary impact of immune checkpoint inhibition on cancer therapy, the lack of response in a subset of patients, as well as the emergence of resistance, remain significant challenges. Here we explore the theoretical consequences of the existence of multiple states of immune cell exhaustion on response to checkpoint inhibition therapy. In particular, we consider the emerging understanding that T cells can exist in various states: fully functioning cytotoxic cells, reversibly exhausted cells with minimal cytotoxicity, and terminally exhausted cells. We hypothesize that inflammation augmented by drug activity triggers transitions between these phenotypes, which can lead to non-genetic resistance to checkpoint inhibitors. We introduce a conceptual mathematical model, coupled with a standard 2-compartment pharmacometric (PK) model, that incorporates these mechanisms. Simulations of the model reveal that, within this framework, the emergence of resistance to checkpoint inhibitors can be mitigated through altering the dose and the frequency of administration. Our analysis also reveals that standard PK metrics do not correlate with treatment outcome. However, we do find that levels of inflammation that we assume trigger the transition from the reversibly to terminally exhausted states play a critical role in therapeutic outcome. A simulation of a population that has different values of this transition threshold reveals that while the standard high-dose, low-frequency dosing strategy can be an effective therapeutic design for some, it is likely to fail a significant fraction of the population. Conversely, a metronomic-like strategy that distributes a fixed amount of drug over many doses given close together is predicted to be effective across the entire simulated population, even at a relatively low cumulative drug dose. We also demonstrate that these predictions hold if the transitions between different states of immune cell exhaustion are triggered by prolonged antigen exposure, an alternative mechanism that has been implicated in this process. Our theoretical analyses demonstrate the potential of mitigating resistance to checkpoint inhibitors via dose modulation.

## Introduction

The advent of immune checkpoint inhibition therapy has shown the remarkable potential of harnessing the immune system to provide much needed care to patients with multiple tumor types. Within just a decade, drugs targeting programmed death-1 (PD-1) and its ligands (PD-L1 and PD-L2) have been approved for over 20 different indications^1–3^. The mechanism of action of immune checkpoint inhibitors (ICIs) is predicated on reversing the state of immune cell “exhaustion”. Exhausted immune cells typically exhibit a progressive loss of their effector functions, such as ability to proliferate, produce cytokines, and effectively bind to and destroy target cells. This dysfunction is often accompanied by the expression of a checkpoint molecule, such as PD-1, CTLA4, TIM3, LAG3, etc^4^. Checkpoint expression increases the recognition time between an immune cell and its target, or between a cytotoxic cell and an antigen-presenting cell, thereby protecting the host from autoimmunity^5^. By interfering with native signaling between a checkpoint on the immune cell (such as PD-1 on CD8 + T cell) and its native ligand, an ICI prevents the cell from receiving a signal to maintain tolerance and thereby allows it to exercise its cytotoxic function.

**Fig. 1:**
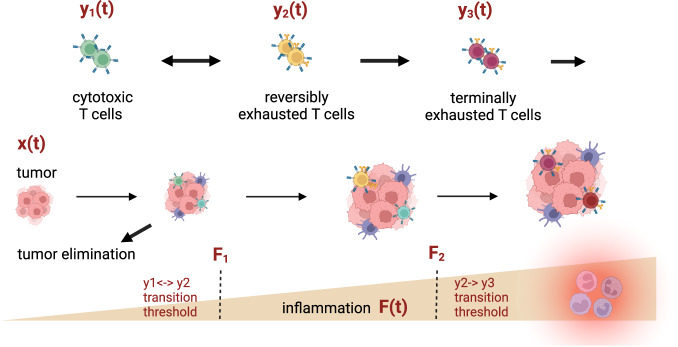
A schematic representation of the impact of inflammation on triggering transitions between different states of immune cell exhaustion. Tumor-immune interactions contribute to an increase in overall state of inflammation *F*. If the tumor is not eliminated by actively cytotoxic T cells, inflammation may surpass a theoretical threshold *F*_1_, triggering transition to reversible exhaustion. If inflammation continues to increase, surpassing a second theoretical threshold *F*_2_, T cells enter a state of terminal exhaustion, which is not susceptible to checkpoint inhibition therapy. As a result, a high degree of inflammation may deplete the pool of reversibly exhausted immune cells, resulting in non-genetic resistance to therapy.

Unfortunately, despite high initial rates of success for some indications, a subset of patients become resistant to ICIs (acquired resistance), while others do not respond from the beginning (primary resistance). Although response rates as high as 70% have been observed in melanoma and Hodgkin’s lymphoma, they are often much lower for most other indications^6^, see also Fig. 1 in Schoenfeld and Hellmann^1^. A number of hypotheses have been proposed for mechanisms underlying both primary and acquired therapeutic resistance to ICIs, including defects in antigen presentation, defects in IFN-gamma signaling, depletion of neo-antigens, among others. For excellent reviews of this topic see, for instance^1,6^,

Another framework for classifying mechanisms of resistance is to categorize them as genetic or non-genetic. Genetic resistance is typically a result of natural selection, where a therapeutic agent, in cancer typically a cytotoxic drug, depletes the population of sensitive cells, leaving behind cells that are resistant to this particular therapy. In such cases, one must switch therapies with the hope of targeting remaining cells from a different angle. Non-genetic resistance instead refers to induction of transient adaptations that may be reversible, and this type of resistance has the potential to be mitigated without switching therapeutic agents.

One example of such a reversible adaptation is described in Hopkins et al. ^7^, where mice treated with PI3K inhibitors developed therapeutic resistance. Upon interrogation, the authors found that administration of PI3K inhibitors evoked a transient diabetic phenotype in the treated mice, producing both hyperglycemia and more importantly hyperinsulinemia. The authors showed that it was not elevated blood sugar that was acting as an additional nutrient source for cancer cells, but high insulin that acted as a growth factor, overcoming the effects of the drug. The authors then treated the mice for hyperinsulinemia using several anti-diabetic drugs including metformin, which reduces glucose production from the liver, and sodium-glucose co-transporter 2 (SGLT2) inhibitors, which increase glucose secretion through urine. A ketogenic diet was also tested as an approach to normalize blood insulin levels. Remarkably, each of these interventions targeting the transient diabetic phenotype restored the animals’ sensitivity to PI3K inhibitors in every tested cell line, including breast, pancreatic, and colorectal cancers, among others^7^. While this example is specific to the metabolism-based mode of action of PI3K inhibitors, it does highlight the existence of reversible phenotypic adaptations that may underlie non-genetic resistance. It also demonstrates how such non-genetic resistance may be mitigated through better understanding of the underlying biology.

Returning to ICIs, growing evidence suggests that immune exhaustion is not a single state. Instead, a T cell can exist in a number of states (likely determined, at least in part, by non-genetic mechanisms), only some of which are susceptible to checkpoint inhibition. In Miller et al. ^8^, both chronic viral infection and tumors were shown to elicit analogous subsets of exhausted CD8 + T cells. The authors described “progenitor exhausted” cells that express checkpoints, such as PD-1, and are thus responsive to checkpoint inhibitor therapy^9,10^, and “terminally exhausted” T cells, which are not susceptible to therapy. In subsequent work, Beltra et al. ^11^ expanded on this framework and proposed a four-cell-stage developmental framework for exhausted CD8 + T cells: quiescent resident, proliferative circulating, circulating mildly cytotoxic, and terminally exhausted resident cells, with each subset defined by specific molecular, transcriptional, and epigenetic characteristics. As such, it appears that the process of T cell exhaustion can broadly be functionally described as T cells transitioning from an active cytotoxic state to a reversible intermediate exhausted state characterized by increased checkpoint expression, to an irreversible terminally exhausted state.

It is not entirely clear what specific factor or likely combination of factors trigger the transition between states. However, if one looks at exhaustion as being an adaptive immune mechanism during chronic infection that most likely evolved to mitigate the development of autoimmunity, it is possible that one such trigger would be the overall inflammatory response that results from cytotoxic activity of the immune cells. In this case, inflammatory cytokines, such as interferons, interleukins, tumor necrosis factors, among others^12^, may accumulate as a result of cytotoxic activity. These cytokines could act as a signal that the costs of prolonged cytotoxic activity are becoming too great, prompting transitions between different states of CD8 + T cell exhaustion.

Within this framework, the following scenario is possible: as the tumor grows and elicits an immune response, it can either be eliminated, controlled, or can progress^13–15^. Due to inflammation triggered by interactions between the tumor and the immune system, some threshold level of tolerance may be surpassed. This threshold is likely determined by a combination of both the levels of inflammatory cytokines and possibly the duration of exposure to them. The surpassing of the threshold is similar to the signaling seen in chronic infections, where an increase in the expression of checkpoints like PD-1 increases recognition time between immune cells and their targets. This process establishes a state of non-genetic and reversible exhaustion. While this can serve the purpose of mitigating autoimmune damage, in the case of cancer it can allow a tumor to continue growing. This further increases inflammation, thereby triggering T cell transition to terminal exhaustion. These mechanisms are summarized in Fig. 1.

Checkpoint inhibitors appear to target only the intermediate reversibly exhausted pool of immune cells^8^. However, if the pool of these cells cannot replenish in time for the next dose, or if the ongoing inflammatory response continues, then it is possible that the newly exhausted immune cells might spend very little time in the intermediate state of reversible exhaustion and rapidly transition to the terminally exhausted state. Consequently, checkpoint inhibitors would lose efficacy not because of genetic resistance, but as a result of incorrect timing of the therapy that does not take into account the broader underlying dynamics of immune cell exhaustion.

Here, we explore this hypothesis using a conceptual mathematical model of inflammation-mediated multi-stage immune cell exhaustion. We use this model to study the effects of altering the timing and dosing of a simulated non-specific checkpoint inhibitor on the outcome of tumor-immune interactions. We show that more frequently administered doses of the same drug can be more efficacious than larger less frequent doses because of the effect on the composition of the immune cell population with respect to state of exhaustion. We conclude with a description of experiments needed to parametrize and validate such a model, and with a discussion of possible implications of this mechanism for mediating non-genetic resistance to immunotherapy.

## Results

In this section, we use the models described in Systems (1) and (2) and parametrized using values in Table 1 to test the hypothesis that changing the treatment protocol (dose and schedule) of the same ICI could mitigate treatment efficacy in a population that is initially ICI-responsive (i.e., does not have primary resistance). We simulate the outputs that would typically be obtained during a mouse experiment, specifically tumor volume and corresponding PK. MATLAB code necessary to reproduce the key results of this paper is available at https://github.com/jgevertz/T_Cell_Exhaustion.

**Table 1:** A summary of parameters used to simulate the model in Systems (1) and (2).

Parameter	Description	Value
${\boldsymbol{\lambda }}$	Tumor growth rate (d^−1^)	0.025
${\boldsymbol{K}}$	Tumor carrying capacity (vol)	3000
${\boldsymbol{b}}$	Rate of tumor cell kill by ${y}_{1}$ (d^−1^vol^−1^)	0.09
${\boldsymbol{c}}$	Rate of tumor cell kill by ${y}_{3}$ (d^−1^vol^−1^)	0.01
${{\boldsymbol{\xi }}}_{{\boldsymbol{1}}}$	Half-maximal rate of tumor cell kill by ${y}_{1}$	1
${{\boldsymbol{\xi }}}_{{\boldsymbol{3}}}$	Half-maximal rate of tumor cell kill by ${y}_{3}$	1
${{\boldsymbol{b}}}_{{\boldsymbol{1}}}$	Rate of ${y}_{1}$ expansion as a result of tumor cell kill by ${y}_{1}$ (d^−1^ vol^−1^)	0.1
${{\boldsymbol{c}}}_{{\boldsymbol{1}}}$	Rate of ${y}_{1}$ expansion as a result of tumor cell kill by ${y}_{3}$ (d^−1^ vol^−1^)	0.001
${{\boldsymbol{d}}}_{{\boldsymbol{1}}}$	Natural clearance rate of ${y}_{1}$ (d^−1^)	0.044
${{\boldsymbol{g}}}_{{\boldsymbol{1}}}$	Rate of transition from active immune cell phenotype ${y}_{1}$ to reversibly exhausted immune phenotype ${y}_{2}$ (d^−1^ vol^−1^)	0.045
${{\boldsymbol{F}}}_{{\boldsymbol{1}}}$	Threshold of inflammatory signaling for transition between active immune cell phenotype ${y}_{1}$ to reversibly exhausted immune phenotype ${y}_{2}$; ${F}_{1} > {F}_{2}$ (vol)	10
${{\boldsymbol{F}}}_{{\boldsymbol{2}}}$	Threshold of inflammatory signaling from reversibly exhausted phenotype ${y}_{2}$ to terminally exhausted immune phenotype ${y}_{3}$; ${F}_{1} > {F}_{2}$ (vol)	25
${{\boldsymbol{k}}}_{{\boldsymbol{d}}}$	Rate of drug-mediated transition from reversibly exhausted immune cell phenotype ${y}_{2}$ to active immune cell phenotype ${y}_{1}$ (d^−1^vol^−1^)	0.001
${{\boldsymbol{g}}}_{{\boldsymbol{2}}}$	Rate of transition from reversibly exhausted immune cell phenotype ${y}_{2}$ back to active immune cell phenotype ${y}_{1}$(d^−1^vol^−1^)	0.01
${{\boldsymbol{g}}}_{{\boldsymbol{3}}}$	Rate of transition from reversibly exhausted immune cell phenotype ${y}_{2}$ to terminally exhausted phenotype ${y}_{3}$(d^−1^vol^−1^)	0.05
${{\boldsymbol{d}}}_{{\boldsymbol{3}}}$	Natural death rate of terminally exhausted immune cells ${y}_{3}$ (d^−1^)	0.01
${{\boldsymbol{b}}}_{{\boldsymbol{2}}}$	Rate of increase of inflammatory factors $F$ in response to cytotoxic activity of ${y}_{1}$ and ${y}_{3}$	0.02
${{\boldsymbol{d}}}_{{\boldsymbol{4}}}$	Natural clearance rate of inflammatory factors $F$ (d^−1^)	0.01
${{\boldsymbol{V}}}_{{\boldsymbol{1}}}$	Volume of distribution, central compartment (mL/kg)	70
${{\boldsymbol{V}}}_{{\boldsymbol{2}}}$	Volume of distribution, peripheral compartment (mL/kg)	33
${\boldsymbol{C}}{{\boldsymbol{l}}}_{{\boldsymbol{1}}}$	Clearance, central compartment (mL/kg/d)	20
${\boldsymbol{C}}{{\boldsymbol{l}}}_{{\boldsymbol{2}}}$	Clearance, peripheral compartment (mL/kg/d)	22
${{\boldsymbol{k}}}_{{\boldsymbol{01}}}$	Drug absorption rate for subcutaneous (sc) administration (d^−1^)	0.11
${{\boldsymbol{k}}}_{{\boldsymbol{10}}}$	Rate of drug clearance from the central compartment (d^−1^) computed as *Cl*_1_/*V*_1_	$C{l}_{1}/{V}_{1}$
${{\boldsymbol{k}}}_{{\boldsymbol{12}}}$	Rate constant for drug distribution from central to peripheral compartment (d^−1^) computed as *Cl*_2_/*V*_1_	$C{l}_{2}/{V}_{1}$
${{\boldsymbol{k}}}_{{\boldsymbol{21}}}$	Rate constant for drug distribution from peripheral to central compartment (d^−1^) computed as *Cl*_2_/*V*_2_	$C{l}_{2}/{V}_{2}$
${\boldsymbol{x}}{\boldsymbol{(}}{\boldsymbol{0}}{\boldsymbol{)}}$	Initial condition for tumor size (vol)	50
${{\boldsymbol{y}}}_{{\boldsymbol{1}}}{\boldsymbol{(}}{\boldsymbol{0}}{\boldsymbol{)}}$	Initial condition for active immune cell phenotype (vol)	0.1

**Fig. 2:**
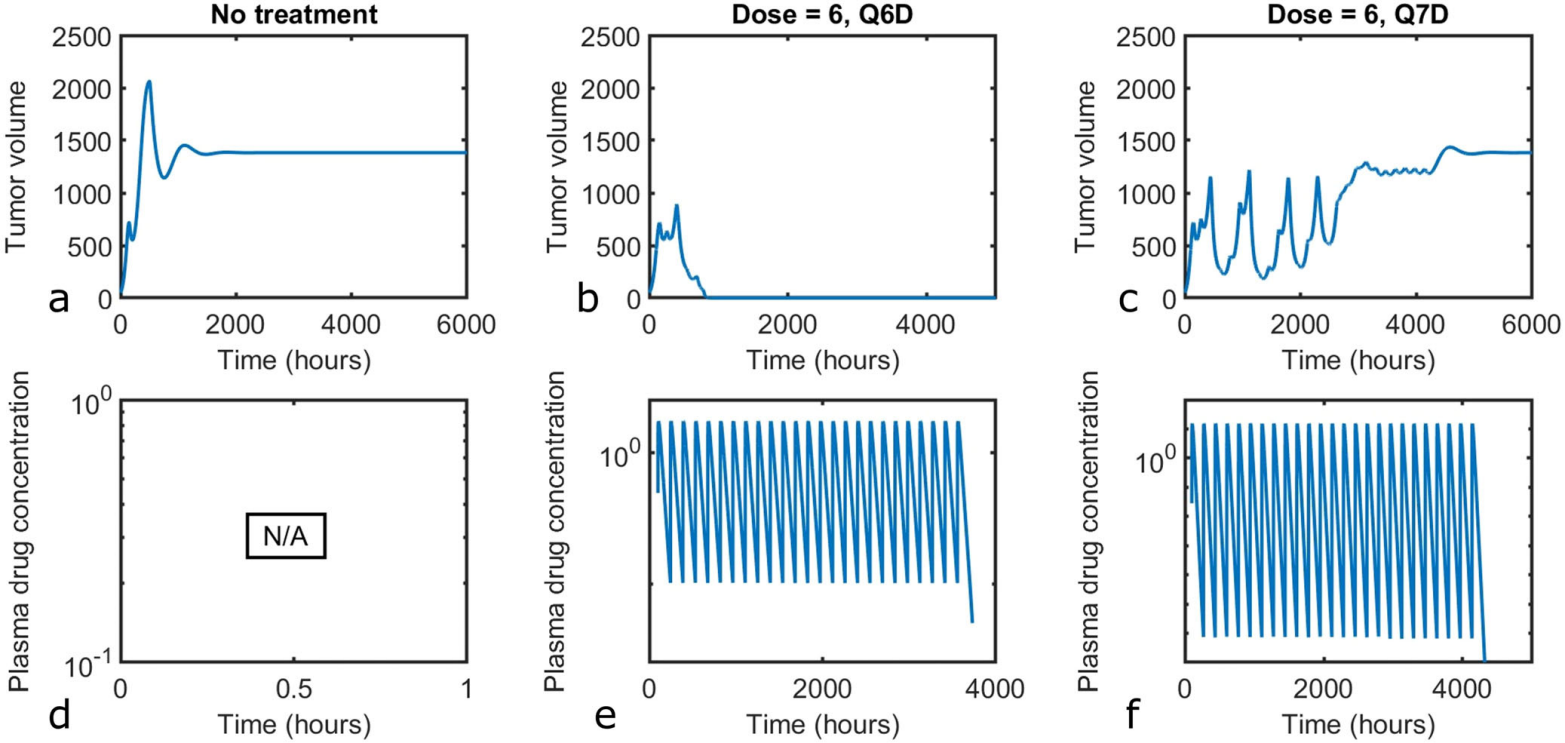
Baseline model behavior. Model simulations predict tumor volume for (**a**) no drug, (**b**) when a dose of 6 units is administered every 6 days (Q6D) for a total of 25 doses, and (**c**) when a dose of 6 units is administered Q7D for a total of 25 doses. Corresponding PK profiles are shown in (**d**) no drug, (**e**) dose of 6 given Q6D, 25 total doses, and (**f**) dose of 6 given Q7D, for 25 total doses.

In Fig. 2a, we confirm that with this model parametrization, in the absence of treatment the tumor grows to a steady state value that is approximately half of its carrying capacity (a fact attributable to the activity of the immune system). For this same parametrization, we show that a dose of 6 units of the simulated drug given 25 times every 6 days (Q6D) is ineffective (Fig. 2c). However, when the same dose and dose number is administered Q7D, the tumor is eliminated (Fig. 2b). This example demonstrates the importance that the timing of dose administration has on treatment response.

### PK metrics do not predict treatment outcome

To interrogate the mechanism that may underlie the profound impact of such a small change in the timing of dose administration, we first conducted a thorough sweep of the protocol space. We fixed the total drug dose (arbitrarily at 50) and assessed the impact of dose fractionation (that is, the number of doses administered, and the spacing between them) on treatment outcome. For each protocol considered, we also computed the values of three standard PK metrics: area under the drug concentration curve (${AU}{C}_{0-\tau }$, where $\tau$ represents spacing between doses), minimum drug concentration at steady state (${C}_{\min }$) and the average drug concentration at steady state ${C}_{{\rm{avg}}}$ (C_avg_).

**Fig. 3:**
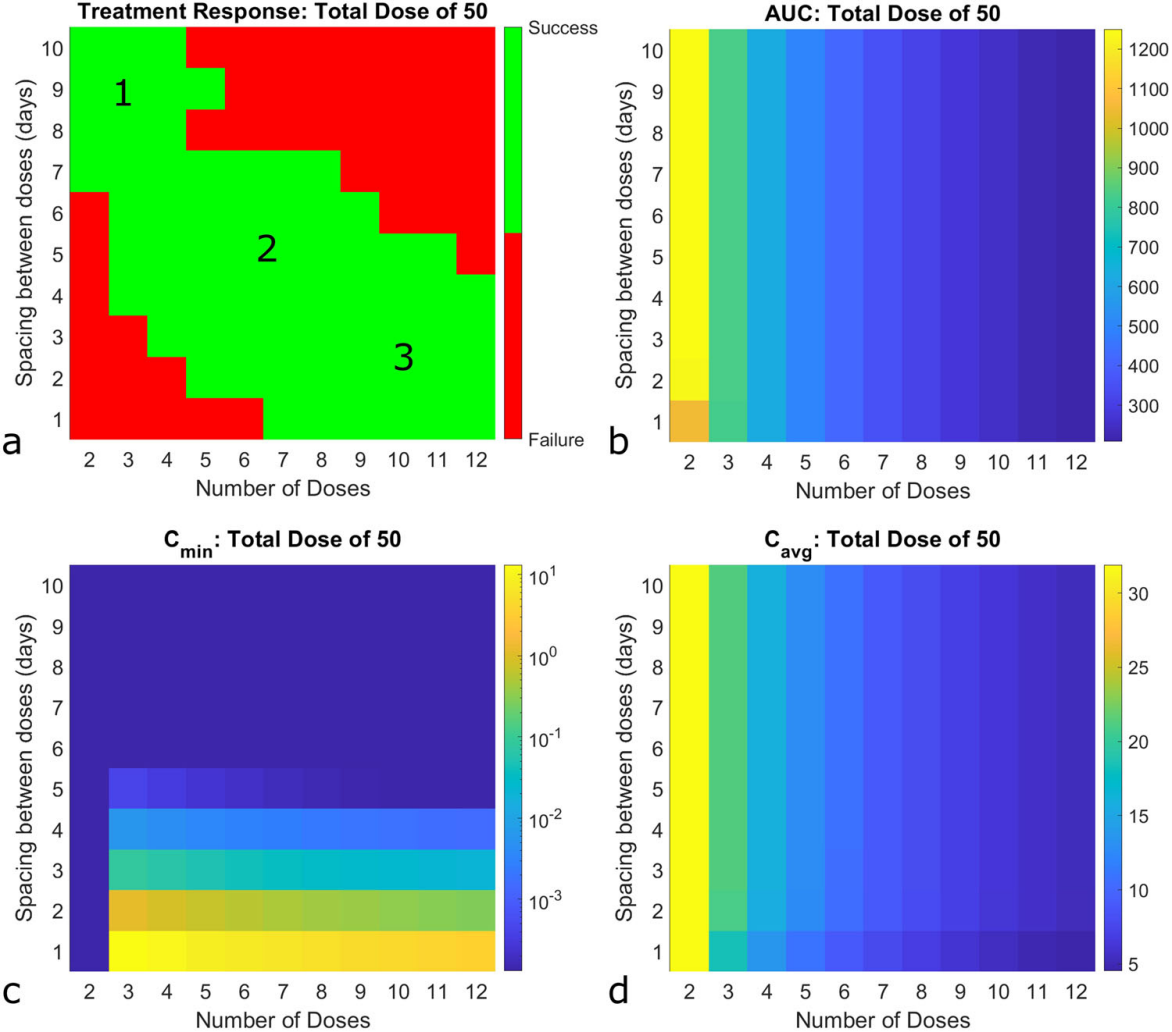
Impact of dose fractionation strategies on treatment outcome. Here the total dose is fixed (arbitrarily at 50), and the effect of fractionating this cumulative dose by number of doses and spacing between them is evaluated. **a** Binary outcome of whether the tumor is eliminated (green) or not (red). **b** Area under the curve (${AU}{C}_{0-\tau }$, where $\tau$ represents spacing between doses), (**c**) Minimum concentration at steady state (${C}_{\min }$) and (**d**) average concentration at steady state (${C}_{{\rm{avg}}}$), corresponding to each dose number-dose spacing combination.

The impact of ICI fractionation on treatment response is shown in Fig. 3a. Interestingly, three protocol designs emerge that can lead to tumor eradication. The first region, labeled with a ‘1’ in Fig. 3a, only administers a small number of doses (two to four doses, with one exception). As the cumulative drug dose is fixed, this means the dose administered each time is large. This “large dose, low dose number” protocol is effective only when the doses are spaced out by a week or more. Interestingly, giving the large doses too close together is not an effective treatment protocol.

In the region labeled with a ‘2’ in Fig. 3a, an intermediate number of doses (three to nine doses) are administered, which results in administering an intermediate drug dose. For this “intermediate dose, intermediate dose number” protocol to be effective, the doses must be administered at least two days apart, but less than 8 days apart. If the doses are too close together, or too far apart, the protocol loses efficacy.

Finally, in the region labeled with a ‘3’ in Fig. 3a, a large number of doses (seven to twelve doses) are administered, which results in administering a small drug dose. For this “low dose, large dose number” protocol to be effective, the doses must be administered sufficiently close together. In particular, the spacing between doses can be no larger than five days. If the doses are given any less frequently, the protocol loses efficacy.

We next asked whether treatment efficacy can be correlated to a standard PK metric, or if it is necessary to consider the output of the entire model to predict treatment efficacy. Somewhat surprisingly, we found that none of the standard PK metrics correlate with efficacy (Fig. 3b–d). For instance, Fig. 3b shows that an ${AU}{C}_{0-\tau }$ value of ~1200, the maximum value achieved, can correspond to both treatment success (for instance, at 2 doses spaced out by 10 days) or treatment failure (for instance, at 2 doses spaced out by 5 days). Similarly, an ${AU}{C}_{0-\tau }$ value of 208, the minimum value achieved, can correspond to both treatment success (for instance, at 11 doses spaced out by 5 days) or treatment failure (for instance, at 11 doses spaced out by 8 days). Figure 3c similarly demonstrates the value of ${C}_{\min }$ does not correlate with treatment efficacy, and Fig. 3d shows how the value of ${C}_{{\rm{avg}}}$ also has no meaningful correlation with efficacy. The absence of any correlation between the various PK metrics and treatment response is further verified in the following cases where the cumulative dose is allowed to vary: 1) the number of doses is fixed, dose and spacing varies (Supplementary Fig. 1) the dose is fixed, number of doses and spacing varies (Supplementary Fig. 2).

We also evaluated the impact of drug half-life on the model predictions. In particular, we repeated the simulations for a drug with a half-life of 9 days rather than the initially simulated 2.6 days. Our results confirm that the three distinct strategies emerge in this model regardless of half-life (albeit the specific projected efficacious dose range is different), and that for a drug with a longer half-life, there remains no meaningful correlation between efficacy and standard PK metrics (Supplementary Fig. 3). Taken together, these results indicate that PK metrics alone are insufficient to predict treatment efficacy.

### Inflammation can play a key role in the effectiveness of checkpoint inhibitors

Given that standard PK metrics cannot predict tumor response in this model, we next selected two representative cases of treatment success and treatment failure to further evaluate the underlying immune dynamics that may affect treatment efficacy.

**Fig. 4:**
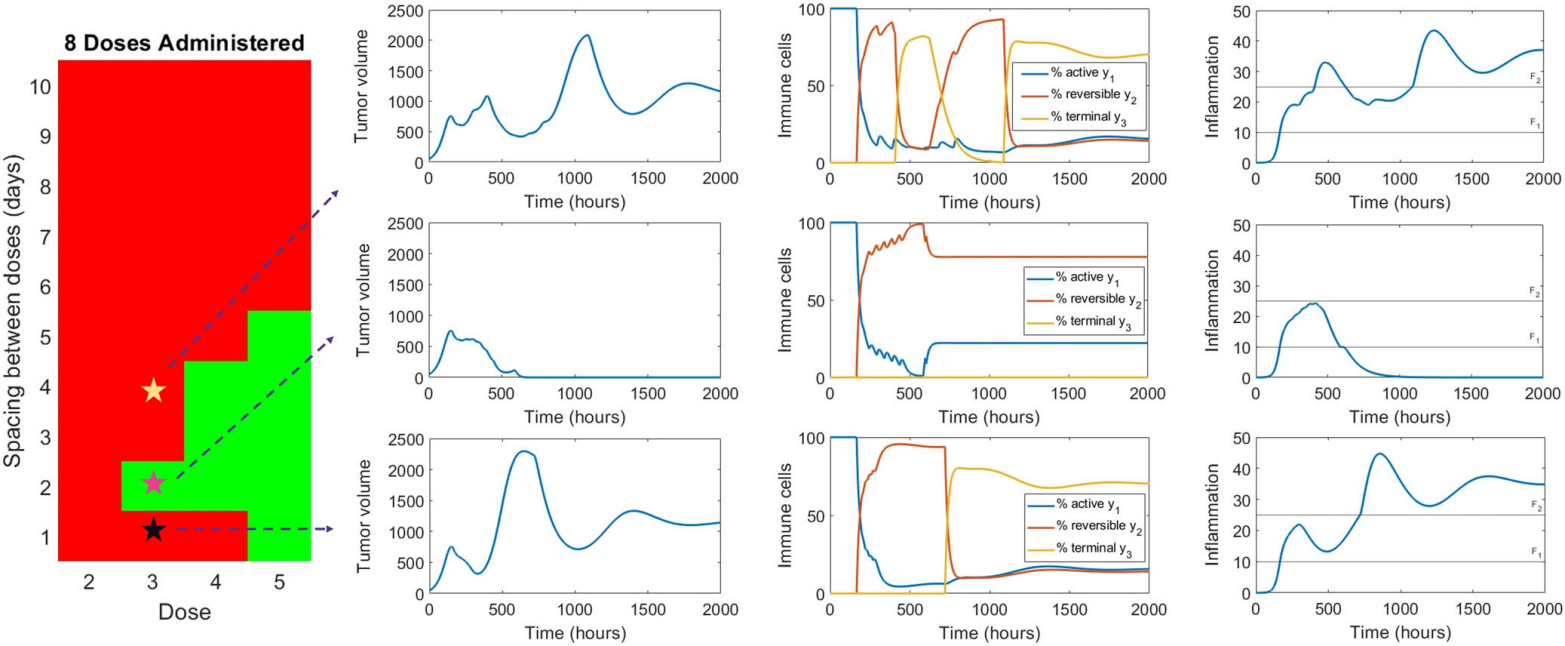
Underlying immune dynamics that determine treatment success or failure. Success is shown in the middle row, where a dose of 3 is given Q2D for a total of 8 doses. Failure is shown when a dose of 3 is given 8 times, either Q4D (top row) or Q1D (bottom row).

As can be seen in Fig. 4, treatment outcome predictably depends on the immune cell composition, and specifically, on whether the immune system composition can maintain a sufficient proportion of actively cytotoxic (${y}_{1}$) and reversibly exhausted (${y}_{2}$) immune cells. This in turn depends on the inflammatory signals that trigger transition particularly from ${y}_{2}$ to ${y}_{3}$ (i.e., from reversibly exhausted to terminally exhausted phenotype), which in this model is represented by the value of parameter ${F}_{2}$. Specifically, if the inflammatory signal is too great (i.e., $F \,>\, {F}_{2}$), then most immune cells transition to the terminally exhausted state. As a result, the drug has too few T cells upon which to act, rendering the treatment ineffective. This phenomenon is shown in Fig. 4, when a dose of 3 is administered 8 times, either Q4D (top panel) or Q1D (bottom panel). Interestingly, the efficacy of the Q1D protocol can be restored through increasing the number of doses, though the same is not true for the Q4D scenario (Supplementary Fig. 2a). On the other hand, if the treatment is timed such that some of the inflammation subsides before the next dose is administered, inflammation $F$ remains largely below ${F}_{2}$. In this case (shown in the middle panel of Fig. 4 at a dose of 3 administered 8 times Q2D), the pool of T cells susceptible to treatment is not depleted, and the treatment is effective. Therefore, within the framework of this model, it is the ability to control inflammation and prevent the ongoing transition to the terminally exhausted phenotype that determines treatment success or failure.

**Fig. 5:**
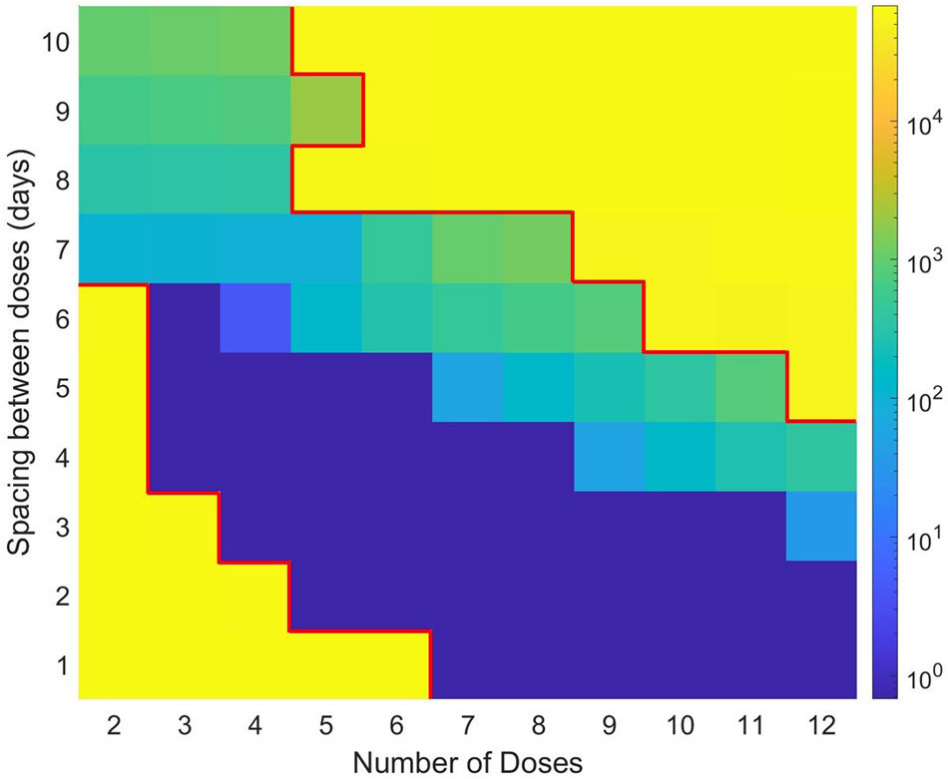
Accumulated inflammation *F* above the threshold for terminal exhaustion, *F*_2_. The dose-schedule protocol sweep considered here is the same as in Fig. 3 (total cumulative dose of 50). Very large values (yellow) indicate that the area accumulated above the ${F}_{2}$ threshold is large, whereas very small values (dark blue) indicate that the area accumulated above the ${F}_{2}$ threshold is small. The red boundary separates the effective protocols (within the boundary) and the ineffective protocols (outside the boundary), as determined in Fig. 3.

To evaluate whether $F\, <\, {F}_{2}$ is indeed necessary to ensure efficacy, we computed the area above the ${F}_{2}$ threshold for the doses and schedules evaluated in Fig. 3. The results, shown in Fig. 5, demonstrate that there is a perfect correlation between the area above the ${F}_{2}$ threshold (which represents a combination of how significantly the threshold was passed, and for how long) and treatment success/failure as shown in Fig. 3a. In particular, all successful treatments have an area over the ${F}_{2}$ threshold of 1976 or less, whereas all ineffective treatments have an area of the ${F}_{2}$ threshold of 53863 or more. Interestingly, for many of the successful protocols that constitute treatment strategies 2 and 3 (intermediate and metronomic-like), the area above ${F}_{2}$ is negligible, meaning that most cells remain in the reversibly exhausted state with little transition to terminal exhaustion. For the borderline protocols and the maximally tolerated dose (MTD)-like strategy, $F$ can surpass ${F}_{2}$ but only transiently; otherwise, protocols lose efficacy.

### Population simulation

We previously saw that three protocol designs can lead to treatment success. The first such design is the maximum tolerated dose (MTD)-like strategy shown in Fig. 3a (region ‘1’), in which a small number of large doses is administered at a low frequency (that is, spaced far apart). The second such design, shown in Fig. 3a (region ‘2’) administers an intermediate number of an intermediate dose of the drug at an intermediate frequency. The third such design is the metronomic-like strategy shown in Fig. 3a (region ‘3’) in which a large number of small doses is administered at a high frequency (that is, they are administered close together). Now, we turn our focus to evaluating treatment effectiveness at the population level, rather than for single model parametrization. We choose to focus this population analysis on the ${F}_{2}$ parameter, as it is likely to be specific to each individual, yet we have demonstrated (Fig. 5) that this parameter (particularly, time spent above this threshold) is predictive of treatment response.

**Fig. 6:**
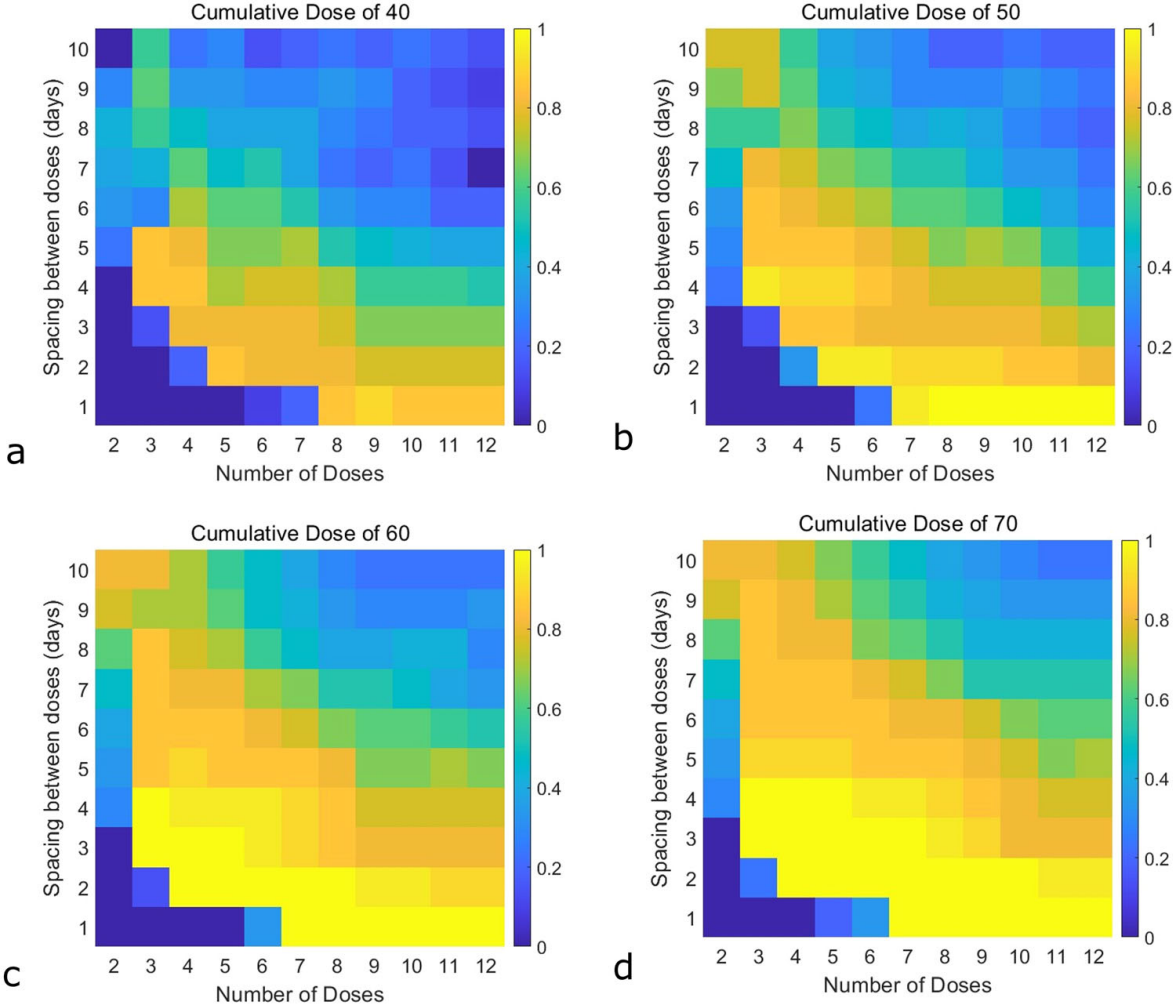
Population-level analysis of immune checkpoint inhibitor efficacy. The proportion of simulated patients that achieve tumor elimination (ranging from 0% eliminated in blue to 100% eliminated in yellow) across a simulated population. This population considers ${F}_{2}$, the inflammatory threshold for transitioning between reversible to terminal exhaustion, taking on integer values [15, 35] with equal probability. Each heatmap shows a fixed cumulative drug dose and fractionates the dose over the specified number of doses (horizontal axis), with variable spacing between doses (vertical axis). Cumulative dose of (**a**) 40, (**b**) 50, (**c**) 60, (**d**) 70.

To conduct our population-level analysis, we record treatment efficacy across protocol space at each integer value of ${F}_{2}$ in the range [15, 35]. This mimics the assumption that ${F}_{2}$ is uniformly distributed over [15, 35], with the mean of the distribution equal to the value of ${F}_{2}$ in Table 1. We think of each value of *F*_2_ as representing a “simulated patient”, and in Fig. 6 we show the probability of treatment success across this ${F}_{2}$ distribution as a function of the cumulative dose administered, and the fractionation of that total dose. As expected, as the cumulative dose increases, a larger set of protocols are associated with treatment efficacy across the simulated patients.

Focusing first on the lowest total dose considered (cumulative dose of 40, Fig. 6a), we find that a metronomic-like protocol (lower doses administered more frequently) maximizes treatment effectiveness across the simulated patients. This particular protocol divides the cumulative dose of the drug over nine doses and spaces the doses out by a single day. It is predicted that nearly 90% of simulated patients are effectively treated by this protocol. Metronomic protocols that divide the cumulative dose over more doses, and keep the spacing fixed at a single day, also achieve over 80% efficacy. Although, it is quite interesting that these more “extreme” metronomic protocols are actually less effective. Metronomic protocols that divide the cumulative dose over less than nine doses can also be effective in 70-80% of simulated patients, but this requires spacing the doses out by two days. A set of “intermediate” protocols (region ‘2’ in Fig. 3a) are also predicted to be effective in around 80% of the simulated population.

When the cumulative dose is increased to 50 (Fig. 6b), there exist protocols that are 100% effective across the simulated population. These protocols are all metronomic-like, dividing the cumulative dose over 8-12 doses, with the doses spaced by a single day. A set of intermediate protocols (larger than the set found at a total dose of 40) is also more than 90% effective at the cumulative dose of 50. The trend observed as we increased from a cumulative dose of 40–50 continues if we consider a total dose of 60 (Fig. 6c) and 70 (Fig. 6d). Some of these more successful protocols are metronomic-like, dividing the cumulative dose into 7-12 doses administered 1–2 days apart. The remainder are “intermediate”, dividing the cumulative dose into 2–7 doses administered 2–4 days apart.

Taken together, these results suggest that the strategy that is most effective for the largest number of individuals is a metronomic-like protocol in which the total drug is administered over a sufficiently large number of doses, with a small spacing between those doses. However, this metronomic-like strategy cannot be taken to an extreme, at least at lower cumulative doses: distributing the cumulative dose over too many doses (as shown in Fig. 6a in the case of 10–12 doses) can actually reduce the probability of treatment efficacy across the population. Non-metronomic-like protocols were also found to be equally effective to metronomic-like protocols at sufficiently high cumulative drug concentrations, though these higher total doses also carry a higher risk of toxicity.

### Sensitivity to model assumptions and parameters

The model analyzed thus far assumes that inflammation levels trigger the transitions between different stages of exhaustion. Another plausible assumption is that excessive and sustained levels of antigen stimulation drive T cell exhaustion, as is seen in some chronic viral infections^11,16,17^. To evaluate whether this alternative mechanism of exhaustion impacts treatment efficacy, we studied a modified version of System (1), where the factor $F$ that drives the transition between T cell phenotypes is instead assumed to represent the level of tumor antigens (see System (3) in the “Methods” section).

To ensure consistency of comparison with the original case we studied, we calibrated the $\bar{{b}_{2}}$ parameter to get inflammatory response on same scale in control case as we saw when cytokines stimulated inflammation (we used $\bar{{b}_{2}}=2.7027\times {10}^{-4})$. We then re-ran the population analysis to assess whether predictions change. Interestingly, while the antigen version of the model has fewer “high-dose, low-frequency” (MTD-like) protocols that are effective (Supplementary Fig. 4), this modification preserved the feature that “low-dose, high-frequency” strategy is still predicted to be successful for the largest proportion of individuals.

**Fig. 7:**
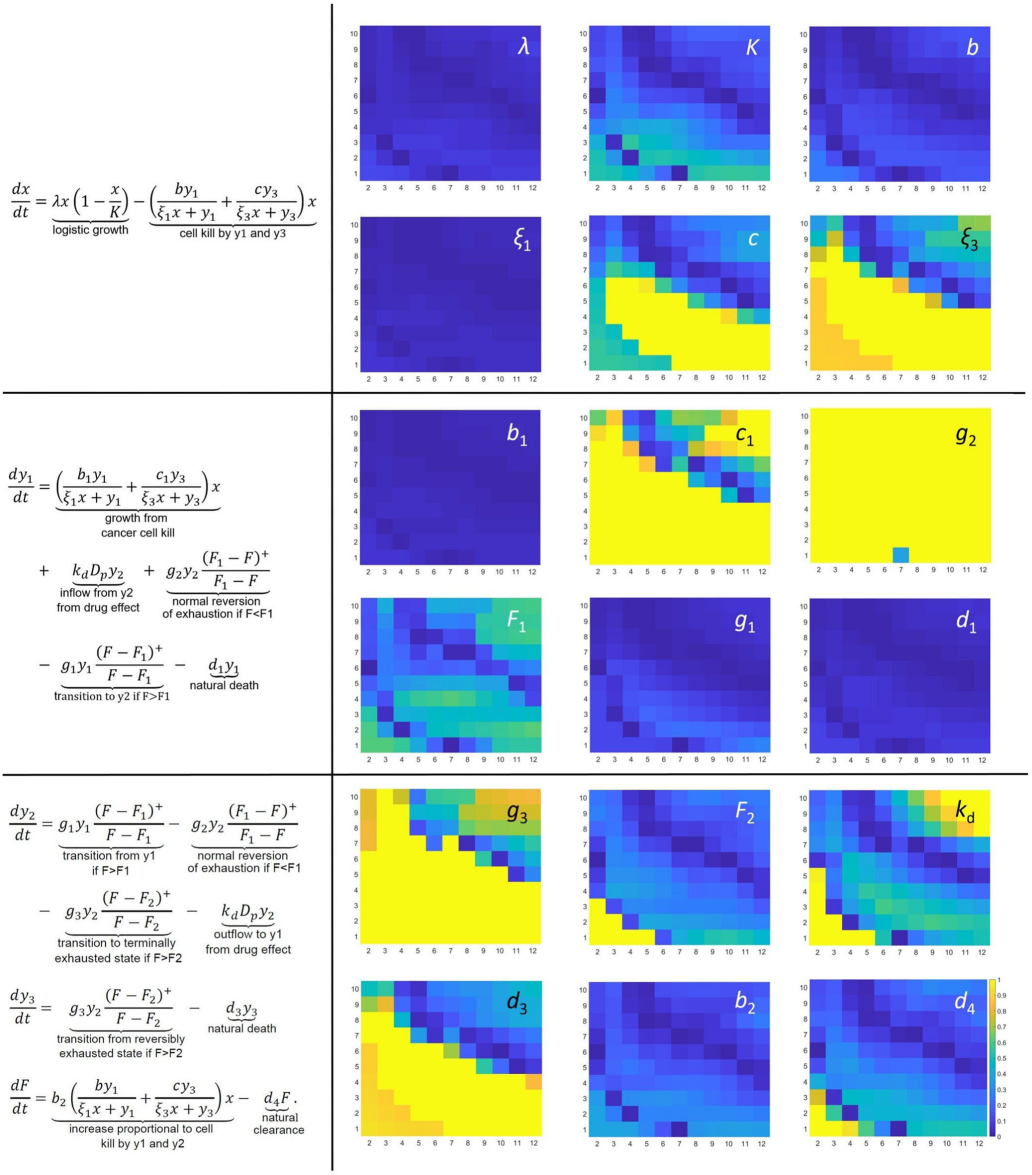
Sensitivity analysis of non-PK model parameters across protocol space. In each heatmap, the horizontal axis indicates the number of doses administered, and the vertical axis is the spacing of the doses (in days). Yellow color represents low sensitivity, blue represents high sensitivity. The cumulative dose administered is fixed at 50, as was used in Figs. 3, 5, and 6b. The heatmap color scale is indicative of the minimal fractional change in the parameter value that “flips” the model prediction on treatment efficacy. The largest fractional change considered was 1 (meaning, a 100% increase and decrease in the default parameter). Thus, any bright yellow value in the heatmap is indicative of the parameter being able to change by at least 100% without flipping the predicting treatment efficacy.

Observing this robustness to the underlying assumption of what drives the transition between T cell phenotypes, we next conducted a parameter sensitivity analysis on the model in Systems (1) and (2) to rigorously quantify the impact of each parameter on treatment efficacy. Specifically, we ask the question: what is the minimal parameter variation (either up or down) that changes the model-predicted efficacy of a specific treatment protocol? In Fig. 7, we answered this question over the full range of treatment protocols considered in Fig. 3. The results are displayed in a set of 18 heatmaps, one for each non-PK model parameter.

A heatmap element in Fig. 7 that is dark blue is indicative of a highly sensitive parameter for a specific protocol: changing the parameter by a small percent reverses the model predictions regarding treatment efficacy (either from success to failure, or vice versa). Figure 7 indicates that there are a number of parameters that the model is highly sensitive across all protocols. Expectedly, the tumor growth rate $\lambda$ is one of these highly sensitive parameters. The other highly sensitive parameters all relate to the behavior of the cytotoxic T cells. These sensitive parameters are their rate of tumor cell kill $b$, their half maximal kill rate ${\xi }_{1}$, their rate of expansion in response to tumor kill by cytotoxic T cells ${b}_{1}$, the rate at which they transition to a reversibly exhausted state ${g}_{1}$, and their natural clearance rate ${d}_{1}$.

In Fig. 7, a heatmap element that is yellow is indicative of a highly insensitive parameter for a specific protocol. We note that the maximum parameter variation considered in our sensitivity analysis is 100%, so any bright yellow value in Fig. 7 is indicative of a parameter that can be varied by at least 100% without changing the model-predicted treatment response. Figure 7 demonstrates that ${g}_{2}$ (transition rate from reversibility exhausted to cytotoxic state) is the only parameter whose value is unimportant in determining treatment response across the effectively all of protocol space (with the exception of 7 doses administered Q1D).

Several parameters stand out as sensitively impacting response in some regions of protocol space, but not in others. These parameters are the cytotoxic capacity of terminally exhausted T cells $c$, the rate of expansion of cytotoxic cells due to cell kill by terminally exhausted cells ${c}_{1}$, the half maximal tumor kill rate by terminally exhausted T cells ${\xi }_{3}$, the rate of transition from reversible to terminal exhausted ${g}_{3}$, the rate of drug-mediated reversion of the exhausted phenotype ${k}_{d}$, and the natural death rate of terminally exhausted cells ${d}_{3}$. Interestingly, the inflammatory threshold for transitioning to terminal exhaustion, ${F}_{2}$, is sensitive over the entirety of protocol space except for when a small number of doses are given close together. Varying ${F}_{2}$ by 100% cannot flip these protocols from ineffective to effective, consistent with what we saw in the population-level analysis in Fig. 6. While ${F}_{2}$ emerges in this analysis as a parameter of intermediate sensitivity, we do note that Fig. 5 demonstrated that the time spent above this threshold perfectly correlates with treatment efficacy.

## Discussion

While genetic (primary or acquired) therapeutic resistance may present an unsurmountable challenge to a particular therapeutic modality, non-genetic resistance has the potential to be mitigated and even reversed if the underlying physiological adaptations are understood^18–20^. Here, we explore a possible mechanism of non-genetic resistance to immune checkpoint inhibition therapy which targets exhausted immune cells. We base our analysis on the emerging understanding that immune cell exhaustion is not a single state but is instead a series of states, only some of which are reversible and therefore targetable by ICIs^8^.

We propose a model that considers transitions between three general immune states: actively cytotoxic, reversibly exhausted, and terminally exhausted, where only reversibly exhausted cells can be targeted by ICIs. We hypothesize that the transition between different states of exhaustion is mitigated by a systemic inflammatory response, since the existence of different states of immune cell exhaustion is most likely an evolutionary adaptation to prevent autoimmunity in chronic infections^21^. We do also evaluate the impact of an alternative mechanism (chronic antigen stimulation) on our predictions. In our analysis, we show that within the framework of this model, none of the standard PK metrics (${C}_{\min }$ and ${C}_{{\rm{avg}}}$ at steady state, or ${AU}{C}_{0-\tau }$) correlate with efficacy. Instead, effectiveness of ICIs is predicated on the level of inflammation, and whether it is maintained beyond the threshold ${F}_{2}$ that signals the transition from the reversibly to the terminally exhausted phenotype. That is, an effective protocol is the one that ensures that inflammation is allowed to fall below ${F}_{2}$ before the next dose is administered, thereby replenishing the pool of reversibly exhausted cells that are susceptible to therapy. This is summarized in Fig. 1. Model simulations at a fixed value of this threshold ${F}_{2}$ suggest that, for a fixed cumulative drug concentration, three types of treatment protocols can result in tumor eradication. The first such design is an MTD-like strategy that divides the total drug into a small number of large doses, administered at a low frequency. The second design divides the total cumulative dose into an intermediate number of doses, administered at an intermediate frequency. The third design is a metronomic-like strategy that divides the total cumulative drug dose into a large number of small doses administered at a high frequency.

Given that these distinct therapeutic designs were effective for a single model parametrization, coupled with the perfect correlation observed between the area above the ${F}_{2}$ threshold and efficacy, we next evaluated whether there is a therapeutic strategy that is predicted to be effective across a population in which the threshold ${F}_{2}$ varies. Interestingly, an MTD-like strategy does not emerge as the optimal treatment design. At the lowest cumulative drug dose that was considered, the most extreme MTD-like strategy is never effective (Fig. 6a, 2 doses spaced 10 days apart). At the highest cumulative drug dose that was considered, this extreme MTD-like protocol is predicted to be nearly 80% effective across the simulated population (Fig. 6d), though the high cumulative dose administered would likely be associated with an increased risk of toxicity.

The lack of optimality of an MTD-like therapy is quite surprising, given that typically ICIs are administered at higher doses every several weeks. For instance, for unresectable or metastatic melanoma, the approved dose of pembrolizumab (Keytruda®) is 200 mg IV Q3W, or 400 mg Q6W^22^. Nivolumab (Opdivo®) is typically administered at 240 mg Q2W or 480 mg Q4W, depending on indication and population^23^. Moreover, re-evaluation of dosing regimens for standard ICIs is typically based on finding regimens that would enable increasing periods between dose administrations, rather than shortening them^24^. Dose selection for these drugs is often directed both by efficacy and by logistics, since it is less disruptive to the patient to receive treatment on a more spaced-out schedule (given acceptable toxicity), and that in turn is enabled by longer half-lives of biologics.

However, based on the underlying mechanism of immune cell exhaustion, our simulations instead indicate the optimality of strategies that give lower doses of the drug more frequently. At all cumulative drug concentrations, except the lowest one considered, such a set of metronomic-like strategies is predicted to be effective across the entire simulated population (Fig. 6b–d). Even at the lowest cumulative drug concentration considered (Fig. 6a), the protocol with the highest probability of success across simulated patients is metronomic-like. Interestingly, a more “extreme” metronomic therapy, where a drug is given daily, is not necessarily better. The model predicts a sweet spot rather than distributing the drug over very many or very few doses.

Clinically, any changes to a dosing strategy must first prove to be non-inferior to existing ones. During the development stage, the highly selective mechanism of action of anti-PD-1 checkpoint inhibitors made it challenging to identify an MTD dose. Therefore, optimal dosing regimens for pembrolizumab, for instance, were determined based on a combination of animal studies, in vitro assays and PK-PD translational models^25,26^. Further analysis of the emergent PK-PD properties of the drug through extensive simulation of anticipated scenarios for potency and PK non-linearity (particularly via target-mediated drug disposition, or TMDD, when the target is not fully saturated) supported the selected dose of 2 mg/kg given Q3W for pembrolizumab^27^. Such a target-engagement-based assessment is typically used as a no regrets strategy, where it is important to ensure that if no efficacy is observed, it is not because the mechanism was not fully engaged through underdosing the patient.

Interestingly, however, in recent years several retrospective analyses were conducted in which a cohort of patients was administered lower than recommended doses of either pembrolizumab or nivolumab for financial reasons. These studies showed that lower doses can be at least as efficacious as MTD. For instance, Chang et al. ^28^ compared the safety and effectiveness of standard (≥ 2 mg/kg) versus low dose (< 2 mg/kg) pembrolizumab with non-small cell lung cancer (NSCLC) in a cohort of 147 patients. They found that both median overall survival (OS) and rate of all classes of immune-related adverse events (irAEs) were similar in both the standard-dose and low-dose pembrolizumab groups. In another retrospective analysis, Low et al. ^29^ evaluated the impact of reducing the dose of pembrolizumab from a 200 to 100 mg flat dose and found no significant difference in response rate or grade 3–4 (severe or life-threatening) irAEs.

Similarly, for nivolumab, Zhao et al. ^30^ recently published a study in which they reviewed the impact of administering low doses of nivolumab to renal cell carcinoma patients. The authors showed that the overall response rate (ORR) was similar in both cohorts, and among the patients in the low dose cohort, one patient had complete response (CR), while no patients had CR in the high-dose cohort. Furthermore, no differences in the number of patients experiencing all-grade or the grade 3–4 irAEs were noted between the two cohorts. While this study has numerous limitations (it was again a retrospective study with a small sample size), it does suggest that lower doses can be at least as efficacious as higher doses in addition to being more affordable^31,32^.

Another important consideration of using lower doses is whether one might be able to mitigate toxicity. In Hurkmans et al. ^33^, the authors analyzed factors that affect PK and its relationship to clinical outcomes in real world patients. They analyzed 588 serum samples derived from 122 advanced-stage cancer patients with several types of cancer, including NSCLC, melanoma and urothelial cell cancer. All patients were treated with pembrolizumab monotherapy either at 2 mg/kg Q3W or 200 mg flat Q3W. The authors evaluated whether individual PK parameters were related to overall survival and irAEs. They reported that severe irAEs (grade 3 or above) were observed in 17% of patients, with the most common ones including gastrointestinal toxicity (8%), immune-related endocrinopathies (3%), hepatotoxicity (3%), pneumonitis (3%), and skin toxicity (3%)^33^. No association was found between pembrolizumab PK and irAEs, with most severe irAEs occurring generally with a notable delay after start of treatment (up to 2 years). These results are consistent with other analyses reporting a lack of statistically significant associations between irAE rates and dose/exposure of anti-PD-1 agents^34^.

Of the reported side effects, delayed hepatic toxicity has been increasingly reported in several case studies^35,36^, including after single-dose administration^37^. While these individual reports require a more thorough investigation, they do suggest that administering some anti-PD-1 checkpoint inhibitors at lower doses might not compromise efficacy but could hold the potential to mitigate both some adverse events, as well as ease the financial burden for patients. In addition, our analysis suggests that altering dosing and scheduling may have the potential additional benefit of mitigating emergence of non-genetic resistance by maintaining a pool of reversibly exhausted T cells that can be targeted by ICIs.

While it may be possible to reduce the doses, there do exist significant logistical challenges to reducing the spacing between them, as this requires increased hospital visits and thus greater demands both on the patient and the hospital staff. While our analysis suggests that a low dose high-frequency strategy would be more likely to work for an ICI given as monotherapy, it may be possible to mitigate such logistical challenges in combination therapy. Specifically, it may be possible to select combination agents that mitigate factors that trigger the transition of T cells from reversible to terminal exhaustion. One approach to do so could be to leverage the sensitivity analysis that has been conducted (Fig. 7), identifying parameters that could contribute most strongly towards changing the treatment outcome (turning an efficacious protocol ineffective and vice versa), and selecting therapeutic agent that may exhibit the properties of altering these sensitive parameters. Then, an optimally synergistic combination strategy can be designed^38^ that preserves the beneficial properties of the spaced out ICI administration schedule while mitigating emergence of non-genetic resistance.

The proposed theory remains to be rigorously validated experimentally, specifically with respect to what triggers the transition between immune phenotypes. Here we propose that it is systemic inflammation, though we also showed robustness in model predictions if the trigger is instead antigen exposure. To assess the assumption that inflammation triggers this transition, a critical experiment would be to find a reasonable range of values for the threshold to terminal exhaustion parameter ${F}_{2}$. This could possibly be done through a standard in vitro co-incubation experiment, where cytotoxic immune cells are co-cultured with different concentrations of a cytokine cocktail, similar to maturation cocktails used for dendritic cells^39^. The expression of specific receptors, such as Ly108 and CD69 reported in Beltra et al. ^11^ as correlated with different states of exhaustion would be assessed to identify typical levels of inflammatory cytokines that functionally trigger a transition between exhaustion phenotypes, thereby benchmarking the level of systemic inflammation that can critically affect therapeutic outcome. Notably, it is possible that there are other factors that trigger this transition, and a more systemic analysis should be conducted to identify such biomarkers, since they may shape the course of treatment. However, if inflammation is indeed an important factor for this transition, it could provide a mechanistic explanation for why high inflammation can be adversely associated with disease outcome^40–43^, with high inflammation potentially weakening the immune response through accelerating the transition of T cells to the state of terminal exhaustion.

An in vivo approach may be more challenging due to limitations of xenograft mouse models, which may develop anti-drug-antibodies (ADA) prior to development of different exhaustion phenotypes. One approach may nevertheless involve conducting parallel experiments, with some animals being treated using the standard MTD-like protocol and others being treated using the proposed metronomic-like protocol and evaluating whether non-genetic resistance emerges, and perhaps whether it can be reversed through change in protocol. To use this experimental setup to parameterize our model, we would require 1) a drug-free control to establish baseline tumor growth, 2) at least two sets of experiments to determine dose-response relationships, and if available, 3) information about immune cell profiling with respect to markers of reversible versus terminal exhaustion, such as CD69 and Ly108, as was shown by Beltra et al. ^11^ Given the likely costs and challenges associated with collecting such detailed information, one could use methodology for minimally sufficient experimental design^44^ to determine at which time points data should be collected for sufficiently informing the mathematical model. However, even in the absence of this level of detail, the proposed in vivo experiments can nevertheless be conducted, although the mathematical model would need to be simplified to eliminate sources of error if some parameters cannot be reasonably estimated. Even in this case, the model-based analysis developed herein revealed a testable hypothesis to be evaluated experimentally.

It should also be noted that while the metronomic-like low dose high frequency approach suggested by this analysis is similar to metronomic therapy for chemotherapeutic agents^45,46^, the underlying mechanism of action is different. Chemotherapy targets cancer cells directly but can also have off-site effects on the immune system, both by ablating cytotoxic cells (thereby hindering therapeutic efficacy), and by acting in an immunogenic manner^46–48^. Other arguments have also been made for reducing the likelihood of genetic resistance emerging through killing therapy-sensitive cells^49,50^, as well as lowering toxicity, which dramatically improves patient quality of life. In contrast, metronomic-like immunotherapy is proposed here to mitigate emergence of non-genetic resistance by allowing for continuous replenishment of therapy-sensitive cells through understanding the impact of collateral inflammation.

More broadly, this reflects an expanding approach to treating cancer as a systemic disease. Chemotherapeutic treatments are geared towards targeting cancer cells directly, with variable efficacy and high toxicity. Changing the timing and scheduling of chemotherapy has allowed mitigating some of the unintended effects^46–48,51^, increasing its effectiveness in some cases. Immunotherapy steps back to augment the power of immune cells that then target cancer, a possibility that has finally been unlocked through the discovery of immune checkpoints^52^. While transformational, it is unfortunately still effective in only a subset of patients^53^. A combination of these two modalities provides an exciting avenue for further improvement of patient outcomes^54,55^. However, perhaps it is now time to step even further back to factor in the systemic effect of therapy on treatment strategy design, where a true combination treatment affects not only cancer cells and its natural immune predator but also other indirect factors that may hinder efficacy.

## Methods

We first describe the dynamics of immune cell exhaustion resulting from interactions with a tumor in the absence of therapy.

We assume the existence of three types of immune cells: fully functional cells ${y}_{1}$, reversibly exhausted cells ${y}_{2}$, and terminally exhausted cells ${y}_{3}$. We also assume that there exists some external factor or combination of factors $F$, which signals the degree of inflammation. If this signal is sufficiently high (when $F$ is greater than some threshold ${F}_{1}$), it triggers the transition from ${y}_{1}$ to ${y}_{2}$. An even higher signal (when $F$ is greater than some threshold ${F}_{2}\, >\, {F}_{1}$) triggers the transition from ${y}_{2}$ to ${y}_{3}$. We assume that these thresholds signal a potential transition to autoimmunity, and thereby exist to slow down the immune response to mitigate this risk. We also assume that ${y}_{2}$ can transition back to ${y}_{1}$ due to the reversible nature of this state of exhaustion, but that terminally exhausted phenotype ${y}_{3}$ cannot transition back to ${y}_{2}$ and is only cleared naturally. A schematic representation of these processes is given in Fig. 1.

We assume that tumor $x$ grows logistically and can be killed by cytotoxic T cells ${y}_{1}$ in a ratio-dependent way (i.e., proportionally to the ratio of immune to cancer cells^56–58^). Further, we assume that, to a lesser extent, tumor cells can be killed by terminally exhausted T cells ${y}_{3}$, while reversibly exhausted cells ${y}_{2}$ have been shown to contribute kill cancer cells “no more efficiently than did naive CD8 + T cells, indicating that they contribute little or no direct cytotoxicity in the TME”^8^. A checkpoint inhibitor, whose dynamics are described using a standard pharmacometrics (PK) model, is assumed to only act on ${y}_{2}$, since, as suggested by^8^, ICIs are only effective against reversibly exhausted immune cells

Finally, the inflammatory factor (or combination of factors) $F$ is assumed to increase proportionally to cancer cell kill by immune cells and undergo natural clearance. The resulting system of equations is as follows:

1 $$\begin{array}{l} \displaystyle\frac{dx}{dt}=\underbrace{\lambda {x}\left(1-\frac{x}{K}\right)}_{{\rm{logistic}}\,{\rm{growth}}}-\underbrace{\left(\frac{b{y}_{1}}{{\xi}_{1}x+{y}_{1}}+\frac{c{y}_{3}}{{\xi}_{3}x+{y}_{3}}\right)x}_{{\rm{cell}}\,{\rm{kill}}\,{\rm{by}}\,{\rm{y}}1\,{\rm{and}}\,{\rm{y}}3}\\\displaystyle\frac{d{y}_{1}}{dt}=\underbrace{x\left(\frac{{b}_{1}{y}_{1}}{{\xi}_{1}x+{y}_{1}}+\frac{{c}_{1}{y}_{3}}{{\xi}_{3}x+{y}_{3}}\right)}_{{\rm{growth}}\,{\rm{from}}\atop{\rm{cancer}}\,{\rm{cell}}\,{\rm{kill}}}+\underbrace{{k}_{d}{D}_{p}{y}_{2}}_{{\rm{inflow}}\,{\rm{from}}\,{\rm{y}}2\atop{\rm{from}}\,{\rm{drug}}\,{\rm{effect}}}+\underbrace{{g}_{2}{y}_{2}\frac{{\left({F}_{1}-F\right)}^{+}}{{F}_{1}-F}}_{{\rm{normal}}\,{\rm{reversion}}\atop{\rm{of}}\,{\rm{exhaustion}}\,{\rm{if}}\,{\rm{F}}\,<\,{\rm{F}}1}-\underbrace{{g}_{1}{y}_{1}\frac{{\left(F-{F}_{1}\right)}^{+}}{F-{F}_{1}}}_{{\rm{transition}}\,{\rm{to}}\,{\rm{y}}2\,{\rm{if}}\,{\rm{F}}\,>\,{\rm{F}}1}-\underbrace{{d}_{1}{y}_{1}}_{{\rm{natural}}\,{\rm{death}}}\\\displaystyle\frac{dy_{2}}{dt}=\underbrace{g_{1}y_{1}\frac{\left(F-F_{1}\right)^{+}}{F-F_{1}}}_{{{\rm{transitoin}}\,{\rm{from}}\,{\rm{y}}1}{{\rm{if}}\,{\rm{F}}\,>\,{\rm{F}}1} }-\underbrace{g_{2}y_{2}\frac{\left(F_{1}-F\right)^{+}}{F_{1}-F}}_{{{\rm{normal}}\,{\rm{reversion}}}{{\rm{of}}\,{\rm{exhaustion}}\,{\rm{F}}\,<\,{\rm{F}} 1} }-\underbrace{g_{3}y_{2}\frac{\left(F-F_{2}\right)^{+}}{F-F_{2}}}_{{{\rm{transition}}\,{\rm{to}}\,{\rm{terminally}}}{{\rm{exhausted}}\,{\rm{state}}\,{\rm{if}}\,\,{\rm{F}}\,>\,\,{\rm{F}}2}} - \underbrace{k_{d}D_{p}y_{2}}_{{{\rm{outflow}}\,{\rm{to}}\,{\rm{y}}1}{{\rm{from}}\,{\rm{drug}}\,{\rm{effect}}}} \\\displaystyle\frac{d{y}_{3}}{dt}=\underbrace{{g}_{3}{y}_{2}\frac{{\left(F-{F}_{2}\right)}^{+}}{F-{F}_{2}}}_{{\rm{transition}}\,{\rm{from}}\,{\rm{reversibly}}\atop {\rm{exhausted}}\,{\rm{state}}\,{\rm{if}}\,{\rm{F}} \,>\, {\rm{F}}2}-\underbrace{{d}_{3}{y}_{3}}_{{\rm{natural}}\,{\rm{death}}} \\ \displaystyle\frac{dF}{dt}=\underbrace{{b}_{2}\left(\frac{b{y}_{1}}{{\xi }_{1}x+{y}_{1}}+\frac{c{y}_{3}}{{\xi }_{3}x+{y}_{3}}\right)x}_{{\rm{increase}}\,{\rm{proportional}}\,{\rm{to}}\,{\rm{cell}}\atop {\rm{kill}}\,{\rm{by}}\,{\rm{y}}1\,{\rm{and}}\,{\rm{y}}3}-\underbrace{{d}_{4}F.}_{{\rm{natural}}\atop {\rm{clearance}}} \end{array}$$

In System (1), the operation ${x}^{+}$ is defined as follows:

$${x}^{+}=\left\{\begin{array}{l}x,x\,\ge\, 0\\ 0,x \,<\, 0.\end{array}\right.$$

An implicit assumption of the model proposed in System (1) is that the tumor and immune cells are well-mixed. However, it is very likely that the tumor vasculature will impact both immune infiltration and drug access, and that reality deviates from the assumption of well-mixed populations. Such considerations could be built into a spatial model, though constructing such a model would necessitate knowledge of the spatial distribution of different cell types with respect to the tumor geometry. In the absence of such data, we instead propose a model of well-mixed populations. Previous modeling work^59^ suggests that models that assume uniform mixing typically result in more optimistic predictions than models that account for spatial effects. For this reason, it is likely that the inclusion of spatial effects would further restrict the set of protocols classified as effective in our theoretical model that assumes well-mixed populations.

We also consider a standard PK model with subcutaneous ICI administration. In this PK model, ${D}_{{sc}}$ is the drug concentration in the subcutaneous compartment, ${D}_{p}$ is the drug concentration in the plasma (central) compartment, and ${D}_{t}$ is the drug concentration in the peripheral (tissue) compartment. Intravenous administration can be described if needed by setting ${k}_{01}=0$.

2 $$\begin{array}{l}\displaystyle\frac{d{D}_{{SC}}}{{dt}}=-{k}_{01}{D}_{{SC}}\\ \displaystyle\frac{d{D}_{p}}{{dt}}={k}_{01}{D}_{{SC}}-{k}_{10}{D}_{p}-{k}_{12}{D}_{p}+{k}_{21}\frac{{V}_{2}}{{V}_{1}}{D}_{t}\\ \displaystyle\frac{d{D}_{t}}{{dt}}={k}_{12}\frac{{V}_{1}}{{V}_{2}}{D}_{p}-{k}_{21}{D}_{t}\end{array}$$

Parameters and nonzero initial conditions used for simulations are summarized in Table 1. It should be noted that while PK values were taken to reflect typical mouse anti-PD1 values with a half-life of approximately 2.6 days^60^, the PK-PD relationships and the values of other parameters are not calibrated to any actual experimental data. They are provided to enable a qualitative study of the projected treatment response based on the proposed description of the underlying biology of T cell exhaustion.

Finally, for the model modification, where antigen levels $F$ increase at a rate proportional to the tumor volume, rather than to the cell kill by ${y}_{1}$ and ${y}_{3}$, last equation in System (1) is modified to be:

3 $$\frac{dF}{dt}=\underbrace{\overline{{b}_{2}}x}_{\begin{array}{c}{\scriptstyle{\rm{increase}}}\,{\scriptstyle{\rm{proportional}}}\\ {\scriptstyle{\rm{to}}}\,{\scriptstyle{\rm{tumor}}}\,{\scriptstyle{\rm{volume}}}\,\\ {\scriptstyle{\rm{as}}}\,{\scriptstyle{\rm{proxy}}}\,{\scriptstyle{\rm{for}}}\,{\scriptstyle{\rm{antigen}}}\,{\scriptstyle{\rm{stimulation}}}\end{array}}\underbrace{-{d}_{4}F.}_{\begin{array}{c}{\scriptstyle{\rm{natural}}}\\ {\scriptstyle{\rm{clearance}}}\,\end{array}}$$

### Reporting summary

Further information on research design is available in the Nature Research Reporting Summary linked to this article.

### Supplementary information

Supplementary Material

Reporting Summary

## Data Availability

The codes for performing all model simulations are available at Github and can be accessed via this link: https://github.com/jgevertz/T_Cell_Exhaustion.

## References

[1] Schoenfeld AJ, Hellmann MD (2020). Acquired resistance to immune checkpoint inhibitors. Cancer Cell.

[2] Twomey JD, Zhang B (2021). Cancer immunotherapy update: FDA-approved checkpoint inhibitors and companion diagnostics. AAPS J..

[3] Lee JB, Kim HR, Ha S-J (2022). Immune checkpoint inhibitors in 10 years: contribution of basic research and clinical application in cancer immunotherapy. Immune Network.

[4] Pardoll DM (2012). The blockade of immune checkpoints in cancer immunotherapy. Nat. Rev. Cancer.

[5] Wykes MN, Lewin SR (2018). Immune checkpoint blockade in infectious diseases. Nat. Rev. Immunol..

[6] Vesely MD, Zhang T, Chen L (2022). Resistance mechanisms to anti-PD cancer immunotherapy. Annu. Rev. Immunol..

[7] Hopkins BD (2018). Suppression of insulin feedback enhances the efficacy of PI3K inhibitors. Nature.

[8] Miller BC (2019). Subsets of exhausted CD8+ T cells differentially mediate tumor control and respond to checkpoint blockade. Nat. Immunol..

[9] Blackburn SD, Shin H, Freeman GJ, Wherry EJ (2008). Selective expansion of a subset of exhausted CD8 T cells by αPD-L1 blockade. Proc. Natl Acad. Sci..

[10] Huang AC (2017). T-cell invigoration to tumour burden ratio associated with anti-PD-1 response. Nature.

[11] Beltra J-C (2020). Developmental relationships of four exhausted CD8+ T cell subsets reveals underlying transcriptional and epigenetic landscape control mechanisms. Immunity.

[12] Lan T, Chen L, Wei X (2021). Inflammatory cytokines in cancer: comprehensive understanding and clinical progress in gene therapy. Cells.

[13] Teng MW, Galon J, Fridman W-H, Smyth MJ (2015). From mice to humans: developments in cancer immunoediting. J. Clin. Investig..

[14] Dunn GP, Old LJ, Schreiber RD (2004). The three Es of cancer immunoediting. Annu. Rev. Immunol..

[15] Vesely MD, Schreiber RD (2013). Cancer immunoediting: antigens, mechanisms and implications to cancer immunotherapy. Ann. N Y Acad. Sci..

[16] Wherry EJ (2011). T cell exhaustion. Nat. Immunol..

[17] Kahan SM, Wherry EJ, Zajac AJ (2015). T cell exhaustion during persistent viral infections. Virology.

[18] Marine J-C, Dawson S-J, Dawson MA (2020). Non-genetic mechanisms of therapeutic resistance in cancer. Nat. Rev. Cancer.

[19] Salgia R, Kulkarni P (2018). The genetic/non-genetic duality of drug “resistance” in cancer. Trends Cancer.

[20] Smith LK, Sheppard KE, McArthur GA (2021). Is resistance to targeted therapy in cancer inevitable?. Cancer Cell.

[21] Kareva, I. & Brown, J. Evolutionary and ecological perspective on the multiple states of T cell exhaustion. In *The Species Within: Cancer Evolution in The Complex Ecosystem Of The Body* (eds Somarelli, J. & Johnson, N.) Ch. 8 (CRC Press/Taylor & Francis, 2023).

[22] Lala M (2020). A six-weekly dosing schedule for pembrolizumab in patients with cancer based on evaluation using modelling and simulation. Eur. J. Cancer.

[23] Long G (2018). Assessment of nivolumab exposure and clinical safety of 480 mg every 4 weeks flat-dosing schedule in patients with cancer. Ann. Oncol..

[24] Jiang M, Hu Y, Lin G, Chen C (2022). Dosing regimens of immune checkpoint inhibitors: attempts at lower dose, less frequency, shorter course. Front. Oncol..

[25] Ahamadi M (2017). Model-based characterization of the pharmacokinetics of pembrolizumab: a humanized anti–PD-1 monoclonal antibody in advanced solid tumors. CPT: Pharmacometrics Syst. Pharmacol..

[26] Patnaik A (2015). Phase I study of pembrolizumab (MK-3475; anti–PD-1 monoclonal antibody) in patients with advanced solid tumors. Clin. Cancer Res..

[27] Elassaiss-Schaap J (2017). Using model-based “learn and confirm” to reveal the pharmacokinetics-pharmacodynamics relationship of pembrolizumab in the KEYNOTE-001 Trial. CPT: Pharmacometrics Syst. Pharmacol..

[28] Chang K-C, Shao S-C, Chen H-Y, Chan Y-Y, Fang Y-F (2022). Comparative effectiveness and safety of standard-dose and low-dose pembrolizumab in patients with non-small-cell lung cancer: a multi-institutional cohort study in Taiwan. Cancers.

[29] Low JL (2021). Low-dose pembrolizumab in the treatment of advanced non-small cell lung cancer. Int. J. Cancer.

[30] Zhao JJ (2021). Low-dose nivolumab in renal cell carcinoma: a real-world experience. Oncology.

[31] Meriggi F, Zaniboni A, Zaltieri A (2023). Low-dose immunotherapy: is it just an illusion?. Biomedicines.

[32] Ratain, M. J. & Goldstein, D. A. Time is money: optimizing the scheduling of nivolumab. *J. Clin. Oncol.* JCO1800045 **11**, (2018). 10.1200/JCO.18.00045 30148658

[33] Hurkmans DP (2021). Prospective real-world study on the pharmacokinetics of pembrolizumab in patients with solid tumors. J. Immunother. Cancer.

[34] Shulgin B (2020). Dose dependence of treatment-related adverse events for immune checkpoint inhibitor therapies: a model-based meta-analysis. Oncoimmunology.

[35] Imoto K (2019). Clinical features of liver injury induced by immune checkpoint inhibitors in Japanese patients. Can. J. Gastroenterol. Hepatol..

[36] Phan T (2021). Very delayed acute hepatitis after pembrolizumab therapy for advanced malignancy: How long should we watch?. Curr. Oncol..

[37] Vries (2023). Case report: pharmacokinetics of pembrolizumab in a patient with stage IV non–small cell lung cancer after a single 200 mg administration. Front. Oncol..

[38] Gevertz JL, Kareva I (2023). Guiding model-driven combination dose selection using multi-objective synergy optimization. CPT: Pharmacometrics Syst. Pharmacol..

[39] Massa C, Thomas C, Wang E, Marincola F, Seliger B (2015). Different maturation cocktails provide dendritic cells with different chemoattractive properties. J. Transl. Med..

[40] Coussens LM, Werb Z (2002). Inflammation and cancer. Nature.

[41] Wilkie KP, Hahnfeldt P (2017). Modeling the dichotomy of the immune response to cancer: cytotoxic effects and tumor-promoting inflammation. Bull. Math. Biol..

[42] Hou J, Karin M, Sun B (2021). Targeting cancer-promoting inflammation—have anti-inflammatory therapies come of age?. Nat. Rev. Clin. Oncol..

[43] Greten FR, Grivennikov SI (2019). Inflammation and cancer: triggers, mechanisms, and consequences. Immunity.

[44] Kareva, I. & Gevertz, J. L. Minimally sufficient experimental design using identifiability analysis. *bioRxiv*10.1101/2023.10.14.562348 (2023). 10.1038/s41540-023-00325-1 PMC10771435 38184643

[45] Kareva I, Waxman DJ, Klement GL (2015). Metronomic chemotherapy: an attractive alternative to maximum tolerated dose therapy that can activate anti-tumor immunity and minimize therapeutic resistance. Cancer Lett..

[46] Chen C-S, Doloff JC, Waxman DJ (2014). Intermittent metronomic drug schedule is essential for activating antitumor innate immunity and tumor xenograft regression. Neoplasia.

[47] Wu J, Waxman DJ (2014). Metronomic cyclophosphamide schedule-dependence of innate immune cell recruitment and tumor regression in an implanted glioma model. Cancer Lett..

[48] Wu J, Waxman DJ (2018). Immunogenic chemotherapy: Dose and schedule dependence and combination with immunotherapy. Cancer Lett..

[49] Gatenby RA, Silva AS, Gillies RJ, Frieden BR (2009). Adaptive therapy. Cancer Res..

[50] Strobl MA (2021). & others Turnover modulates the need for a cost of resistance in adaptive therapy. Cancer Res..

[51] Biziota E, Mavroeidis L, Hatzimichael E, Pappas P (2017). Metronomic chemotherapy: a potent macerator of cancer by inducing angiogenesis suppression and antitumor immune activation. Cancer Lett..

[52] Shiravand Y (2022). & others Immune checkpoint inhibitors in cancer therapy. Curr. Oncol..

[53] Robert C (2020). A decade of immune-checkpoint inhibitors in cancer therapy. Nat. Commun..

[54] Bailly C, Thuru X, Quesnel B (2020). Combined cytotoxic chemotherapy and immunotherapy of cancer: modern times. NAR Cancer.

[55] Luo Q, others. (2019). Emerging strategies in cancer therapy combining chemotherapy with immunotherapy. Cancer Lett..

[56] Akcakaya HR, Arditi R, Ginzburg LR (1995). Ratio-dependent predation: an abstraction that works. Ecology.

[57] Berezovskaya F, Karev G, Arditi R (2001). Parametric analysis of the ratio-dependent predator–prey model. J. Math. Biol..

[58] Pillis LG, de, Radunskaya AE, Wiseman CL (2005). A validated mathematical model of cell-mediated immune response to tumor growth. Cancer Res..

[59] Gevertz, J. L. et al. Exploring the importance of the microenvironmental niche and tumor heterogeneity through a spatial model. In *Applications of Dynamical Systems in Biology and Medicine, IMA Volumes in Mathematics and its Applications*, Vol. 158 (eds Radunskaya, A. & Jackson, T.) (Springer-Verlag, 2015).

[60] Lindauer A (2017). Translational pharmacokinetic/pharmacodynamic modeling of tumor growth inhibition supports dose-range selection of the anti–PD-1 antibody pembrolizumab. CPT: Pharmacometrics Syst. Pharmacol..

