# Supplementary material for: Mitigating non-genetic resistance to checkpoint inhibition based on multiple states of immune exhaustion
Source: NPJ Syst Biol Appl. 2024 Feb 9;10:14. doi: 10.1038/s41540-024-00336-6 (PMC10858190; doi:10.1038/s41540-024-00336-6)
Supplement: Supplementary file 1 — Supplementary Material [file 41540_2024_336_MOESM1_ESM.pdf]

Mitigating non-genetic resistance to checkpoint inhibition based on multiple states of immune exhaustion

Irina Kareva and Jana L. Gevertz

Supplementary Information

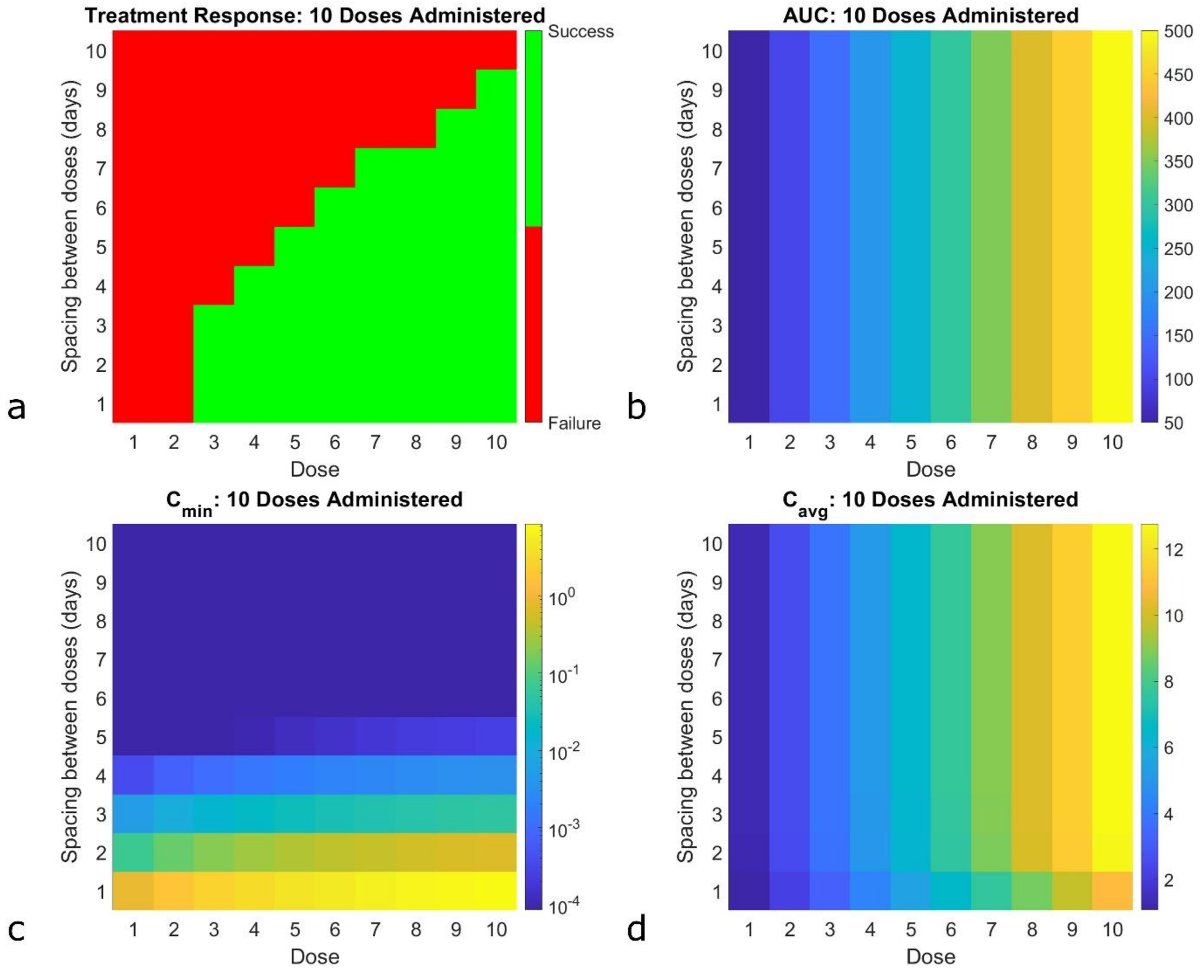

Supplementary Figure 1: Protocol sweep varying both the dose of ICI, and the spacing between doses (frequency of administration) for a fixed number of doses (here 10 doses). (a) Binary outcome of whether the tumor was eliminated (green) or not (red). (b) Area under the curve ($AUC_{0-\tau}$, where τ represents spacing between doses), (c) Minimum concentration at steady state ($C_{\min}$) and (d) average concentration at steady state (C_{avg}), corresponding to each dose amount-frequency combination.

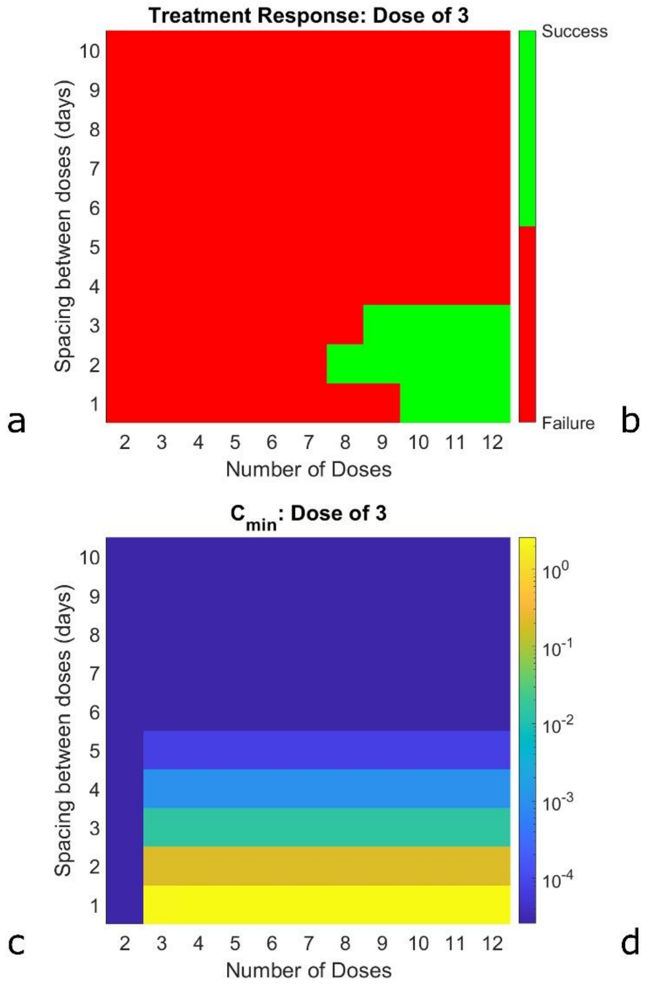

Supplementary Figure 2: Protocol sweep varying both the number of doses of ICI, and the spacing between doses (frequency of administration) for a fixed dose (here a dose of 3). (a) Binary outcome of whether the tumor was eliminated (green) or not (red). (b) Area under the curve ($AUC_{0-\tau}$, where τ represents spacing between doses), (c) Minimum concentration at steady state ($C_{\min}$) and (d) average concentration at steady state (C_{avg}), corresponding to each dose number-frequency combination. (a) indicates that Q1D protocol shown in Figure 4 can be rescued (flipped from ineffective to effective) by increasing the number of doses, but the Q4D protocol cannot.

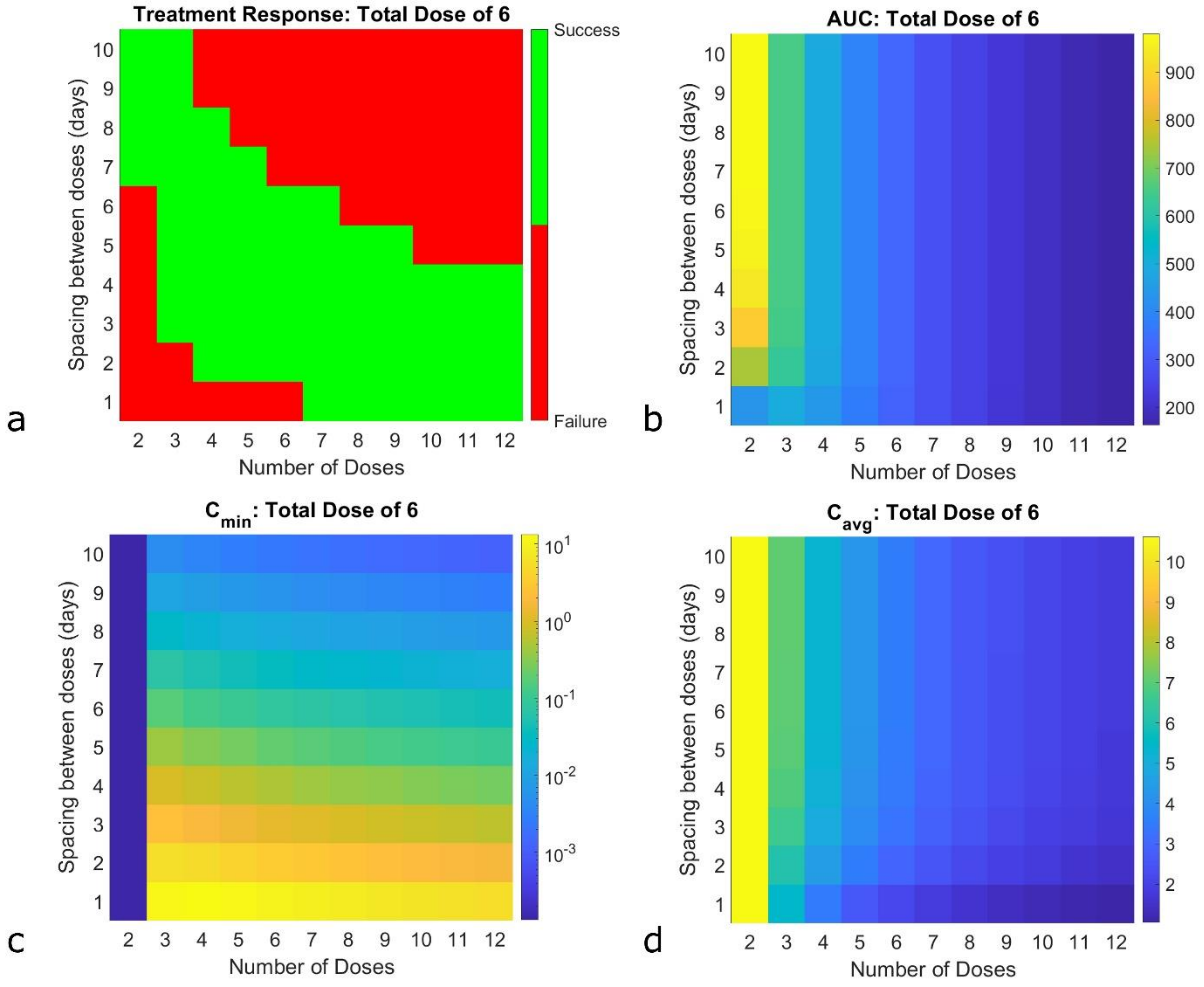

Supplementary Figure 3: Simulation of a drug with a longer half-life. The PK parameters were changed to: V_1 = 39.3 mL/kg, V_2 = 39 mL/kg; Cl_1 = 3.06 mL/kg/d, Cl_2 = 15.65 mL/kg/d. These correspond to a half-life of approximately 9 days. The qualitative model predictions are unchanged compared to a drug with a shorter half-life, and there is still no correlation between efficacy and PK metrics.

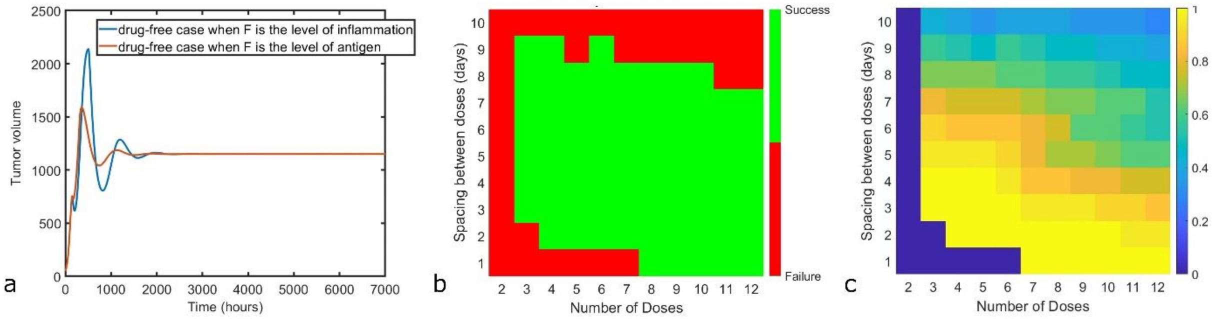

Supplementary Figure 4: Population-level analysis under the assumption that antigen exposure, and not inflammation, triggers transitions between exhaustion stages. (a) model calibration to confirm that treatment-free case in model version 2 (where F represents levels of tumor antigen) behaves in a manner consistent with model version 1 (where F represents inflammation). (b) Impact of dose fractionation strategies for a total dose of 50 (chosen arbitrarily for illustration purposes) for model version 2 is consistent with predictions for model version 1 in the main text. (c) The proportion of simulated patients that achieve tumor elimination (ranging from 0% eliminated in blue to 100% eliminated in yellow) across a simulated population. This population considers the antigen-exposure threshold for transition between reversible to terminal exhaustion taking on integer values [15, 35] with equal probability. The metronomic-like strategy for both models is still predicted to be most effective for the largest fraction of a virtual population.
